# Eruptive Xanthomas as Cutaneous Manifestation of Familial Combined Dyslipidaemia in an Eleven-year-old: A Case Report

**DOI:** 10.31729/jnma.4816

**Published:** 2020-03-31

**Authors:** Anupa Khadka, Sabina Bhattarai

**Affiliations:** 1 Department of Dermatology, Kathmandu Medical College and Teaching Hospital, Sinamangal, Kathmandu, Nepal

**Keywords:** *eruptive xanthoma*, *familial*, *hypercholesterolemia*

## Abstract

Xanthomas are subcutaneous lipid deposits containing macrophages loaded with cholesterol and cholesterol esters. Although quite common in adults, xanthomas in pediatric population are infrequent and when present, may represent a cutaneous manifestation of underlying lipoprotein disorders which most often are familial. We report a case of an eleven-year-old female child, with multiple eruptive xanthomas of skin since two years of age, a positive family history and deranged lipid profile consistent with possible familial hypercholesterolemia.

## INTRODUCTION

Xanthomas are subcutaneous, benign collections of lipids which are histologically characterized by their accumulation in large foam cells within the skin.^1^ Xanthomas can present as an early manifestations of systemic disorders, mainly dyslipidemia or uncommonly a sole manifestation. Early recognition and prompt management of underlying condition significantly decreases morbidity and mortality. We present a case of an eleven-year-old female child with clinical and histopathological picture suggestive of eruptive xanthomas, a positive family history and underlying combined dyslipidemia.

## CASE REPORT

An eleven-year-old non obese female child, born of non-consanguineous marriage presented with multiple, asymptomatic, yellow to brownish lesions over multiple sites including hands, elbows, knees and eyes. Lesions started at 2 years of age and gradually progressed in size and number, initially starting from outer aspect of multiple interdigital web spaces of both hands. Over a span of next 8 years, similar lesions progressively developed over multiple knuckles, palmar creases, both knees, lower aspect of both buttocks and periocular areas respectively. Family history of similar condition was positive in elder sister (14 years of age) and maternal aunt. There was no history of diabetes, cardiac, liver or renal diseases in the patient or any of the family members. Patient had no significant drug intake history. No significant history of loss of weight or appetite, and any relevant systemic symptoms were present.

**Figure 1:**
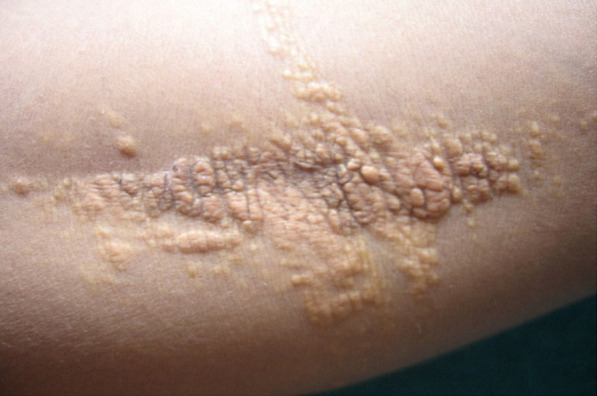
Yellowish papules and plaques with koebnarization on skin crease of right elbow.

**Figure 2:**
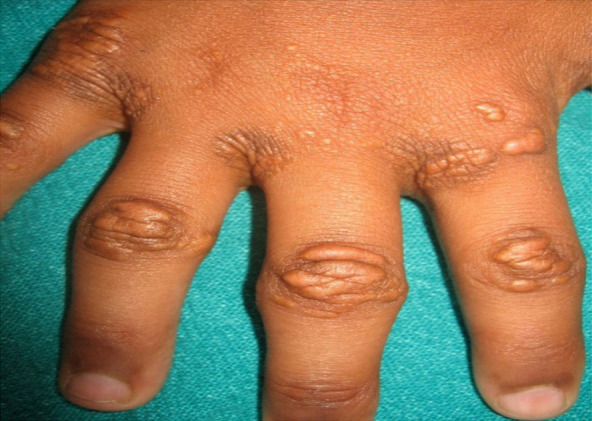
Xanthomas in interdigital spaces and dorsum of inter pharyngeal joints.

**Figure 3:**
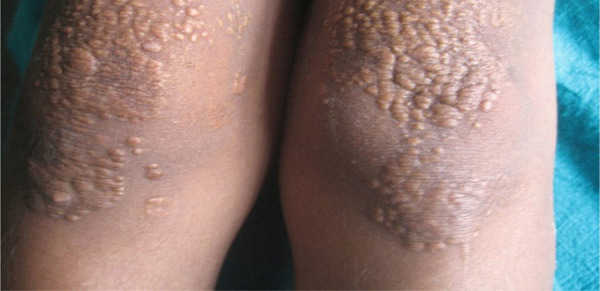
Crops of papular xanthomas on extensor aspect of bilateral knees.

**Figure 4:**
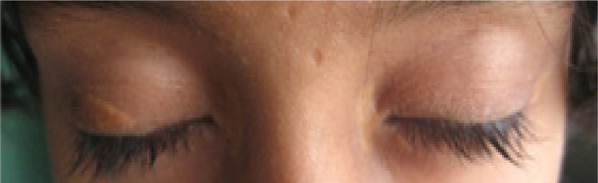
Xanthelama palpebrarum over bilateral eyelids and medial canthi.

Examination revealed crops of multiple, smooth surfaced, yellow to brownish, well defined, circular to irregularly shaped, solitary papules and coalesced plaques, sized (0.2 x 0.2 to 5 x 10) cm^2^ which were bilaterally and symmetrically distributed over flexor aspect of elbow (Figure 1), dorsal aspect of interphalangeal joints and interdigital webs (Figure 2); extensor aspect of knees (Figure 3); upper eyelids, medial and lateral canthi (Figure 4). They were of soft to firm consistency on palpation and some lesions with koebnarization were also present. The child was developmentally appropriate for her age. There was no lymphadenopathy or organomegaly. Systemic examination was normal. Investigations revealed Fasting Lipid Profile (FLP) as, Total serum cholesterol (TC).

**Figure 5:**
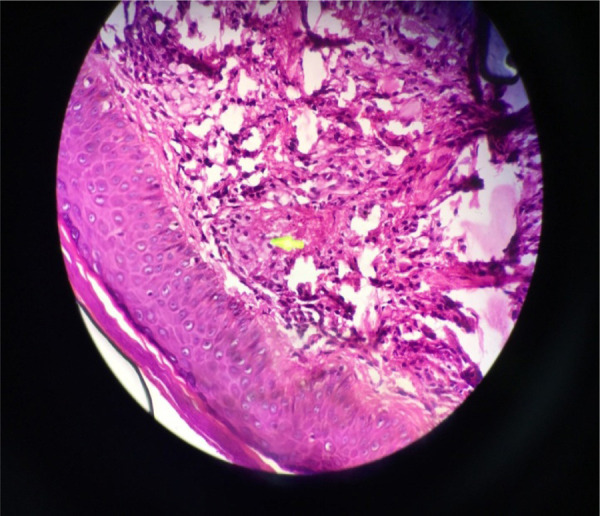
Hematoxylin and Eosin stain, 40 X Magnification shows infiltration of foamy cells within the reticular dermis with admixture of lymphocytes.

769mg/dl, High density lipoprotein (HDL)- 31mg/ dl, Serum triglyceride (TGs)- 316mg/dl, Low Density Lipoprotein (LDL) -176mg/dl. Histopathological examination showed infiltration of foamy cells within the reticular dermis with admixture of lymphocytes (Figure 5). X-ray chest, Ultrasonography of abdomen, ECG, Echocardiogram, Blood sugar, and Liver Function Test (LFT) were all normal.

Based on history of similar symptoms in family members, characteristic clinical findings since early childhood and investigations, a diagnosis of eruptive xanthomas secondary to familial combined hyperlipidemia (a possible Familial Hypercholesterolemia as per Simon Broome Register criteria)2 was made. Patient was managed with dietary modification and oral Atorvastatin 20 mg /day after physician consultation. The parents were counseled about the disorder; and family screening for lipid profile, LFT and cardiac diseases along with regular follow up for child was advised.

## DISCUSSION

Eruptive xanthomas represent sudden eruption of one to four millimetres sized, yellow orange, dome shaped papules, generally distributed over the extensors of extremities, buttocks and hands.^3^

Xanthomas may occur in normolipemic or hyperlipidemic and in some, may be associated with lymphoproliferative disorders, histiocytic disorders and malignancies. A variety of primary genetic disorders, and secondary conditions such as obesity, excessive alcohol intake, diabetes (type 2), drugs (Retinoids, estrogenand protease inhibitors), hypothyroidism, cholestasis, biliary atresia, renal disease, and pancreatitis, or both may be the cause of underlying hyperlipoproteinemia that result not only in xanthomas but also life-threatening vascular atherosclerosis.^4,5^

Clinically, xanthomas can be eruptive, tubo-eruptive or tuberous, tendinous, or planar.^1,6^ Although not specific, morphology of xanthoma may be indicative of underlying type of dyslipidaemia. However, histopathology is characteristic. Eruptive xanthomas exhibit smaller and fewer foam cells, with infiltration of lymphoid cells, histiocytes, neutrophils, and free lipid (predominantly triglyceride) in the dermis.^7^

When xanthomas occur in children and adolescents, as in our case, a more severe form of hyperlipidemia should be suspected.^5^ Homozygous Familial Hypercholesterolemia wherein two abnormal LDL receptor genes are inherited, with resultant defective lipoprotein catabolism, usually presents with cutaneous xanthomas in early childhood and cardiovascular abnormalities in the second or third decade of life. It is characterized by markedly elevated levels of plasma cholesterol, normal to mildly elevated Triglycerides and up to six to eight-fold elevation of plasma LDL.^8^ Although uncommon with a prevalence of one in million,^9^ association of cardiovascular abnormalities makes a full workup on such children imperative.

Laboratory analysis should be performed on all patients with a new diagnosis of eruptive xanthoma with unclear origin to evaluate for causes of hyperlipidaemia. Lack of provision for gene analysis study limited definitive diagnosis of underlying variant of dyslipidaemia in our study; however, genetic counselling and family tracing is important in these patients. Dietary modifications i.e. low-fat diet with appropriate energy content for age and limited daily intake of cholesterol and early institution of pharmacologic treatment for hyperlipidaemia should be considered. HMG COA reductase inhibitors or statins are mainstay of treatment. Combination therapy with ezetimibe, bile acid sequestrants and other cholesterol lowering medications like nicotinic acid and fibrates have less often been used in children.^1,8,10^ Plasmapheresis and adjunctive combination therapy of high-dose statins and ezetimibe is advocated for Homozygous Familial Hypercholesterolemia as most effective whenever available.^10^

Familial cutaneous xanthomas in children are fairly uncommon. While asymptomatic and non-life threatening on their own, eruptive xanthomas can enable identification and early management of associated dyslipidaemias and associated grave complications

**Consent: JNMA Case Report Consent Form** was signed by the patient and the original article is attached with the patient's chart.

## Conflict of Interest

**None.**
